# DNA fingerprinting in zoology: past, present, future

**DOI:** 10.1186/2041-2223-5-3

**Published:** 2014-02-03

**Authors:** Geoffrey K Chambers, Caitlin Curtis, Craig D Millar, Leon Huynen, David M Lambert

**Affiliations:** 1 School of Biological Sciences, Victoria University of Wellington, PO Box 600, Wellington 6140, New Zealand; 2 Environmental Futures Research Institute, Griffith University, Nathan, QLD 4111, Australia; 3 Allan Wilson Centre for Molecular Ecology and Evolution, School of Biological Sciences, The University of Auckland, Auckland 1142, New Zealand

**Keywords:** Multilocus VNTR probes, Single locus probes, Avian mating systems, Microsatellite DNA

## Abstract

In 1962, Thomas Kuhn famously argued that the progress of scientific knowledge results from periodic ‘paradigm shifts’ during a period of crisis in which new ideas dramatically change the *status quo*. Although this is generally true, Alec Jeffreys’ identification of hypervariable repeat motifs in the human beta-globin gene, and the subsequent development of a technology known now as ‘DNA fingerprinting’, also resulted in a dramatic shift in the life sciences, particularly in ecology, evolutionary biology, and forensics. The variation Jeffreys recognized has been used to identify individuals from tissue samples of not just humans, but also of many animal species. In addition, the technology has been used to determine the sex of individuals, as well as paternity/maternity and close kinship. We review a broad range of such studies involving a wide diversity of animal species. For individual researchers, Jeffreys’ invention resulted in many ecologists and evolutionary biologists being given the opportunity to develop skills in molecular biology to augment their whole organism focus. Few developments in science, even among the subsequent genome discoveries of the 21st century, have the same wide-reaching significance. Even the later development of PCR-based genotyping of individuals using microsatellite repeats sequences, and their use in determining multiple paternity, is conceptually rooted in Alec Jeffreys’ pioneering work.

## Introduction

Unique DNA fingerprints arise as a result of restriction enzyme digestion of an individual’s tandem repeat loci. In individuals belonging to sexually outbreeding populations, the resulting multilocus DNA profiles are typically variable and unique to the individual. These multilocus ‘minisatellite’ DNA repeats (also known as Variable Number Tandem Repeats, VNTRs), typically consisting of repeated 10 to 60 bps units, are highly variable in length, as are restriction enzyme fragments, and are commonly detected by hybridization of radiolabeled VNTR probes to restriction enzyme-digested and size-separated genomic DNA. DNA fingerprinting was originally developed as a tool for human identification in forensic investigations [1], and later found application in immigration cases [2] and paternity disputes [3]. Moreover, the subsequent use of DNA profiling to establish the innocence of numerous convicted persons prompted a re-examination of the reliability of eyewitness evidence [4]. The discovery of a genetic system that would uniquely identify a person was unanticipated, even to Jeffreys: ‘We walked out of the darkroom looking at this complicated mess on an X-ray film and thought “Whoa, wait a minute. We’ve stumbled on the potential for DNA-based biological identification”’ [5]. Ideas concerning the potential application of the technique were equally novel. According to Jeffreys: ‘When I talked about it in a Department seminar, and then speculated about what we could use this for, like catching rapists from semen—about a third of the audience fell over laughing. It sounds bizarre now because it’s so blindingly obvious that you can use DNA for this, but believe me, back in the 80s it was simply not there’ [6].

Jeffreys’ technique [7] resulted in a fundamental change in the discipline of zoology as it became apparent that DNA fingerprinting could also be applied to a wide range of bird and other animal species. Within two years from its description [7] and the first application of DNA fingerprinting within a legal context [2], two pioneering reports were published on mating systems in house sparrows (*Passer domesticus*) [8,9]. These were the first of many studies demonstrating the power with which this technique could typically determine the genetic identity of individuals and measure genetic variation in natural populations. This represented a significant advance over the degree of genetic resolution that had been available with earlier isozyme-based techniques, allowing true genetic relationships among individuals to be determined, rather than them being inferred (often erroneously) from field observations. This uncovered some remarkable surprises, such as the finding that many species are characterized by significant levels of extra-pair paternities, and even maternities. One example of this was the discovery that in the Australian superb fairywren (*Malurus cyaneus*), up to 75% of matings consist of extra-pair copulations [10]. DNA fingerprinting has also enabled zoologists to test predictions of kin selection models [11] in a genuinely meaningful way, and multilocus profiles have also been used to detect species hybrids [12]. The same methods have been applied less frequently and less successfully to describe population structure and evolution, though the success increased with the advent of single locus methods. Finally, we note that minisatellite DNA fingerprinting has created spin-off technologies, such as the non-invasive determination of sex [13-15].

Among some zoologists there was a rapid embrace of the technical challenges associated with DNA fingerprinting technology. Scientists from Brazil, Canada, Germany, New Zealand, and the UK featured prominently among the major early contributors. Application of early minisatellite DNA fingerprinting tools rapidly progressed through three stages of development: multilocus minisatellite probes, single locus minisatellite probes, and digital array minisatellite typing. These techniques were applied to a wide range of species and ecological questions, some of which will be covered in this review. The majority of the minisatellite work was carried out and reported from 1987 to 1994 with a trickle of ‘heritage’ reports published as late as 2000 [16].

## Review

### Technical aspects of minisatellite DNA fingerprinting methodology

**Figure 1:**
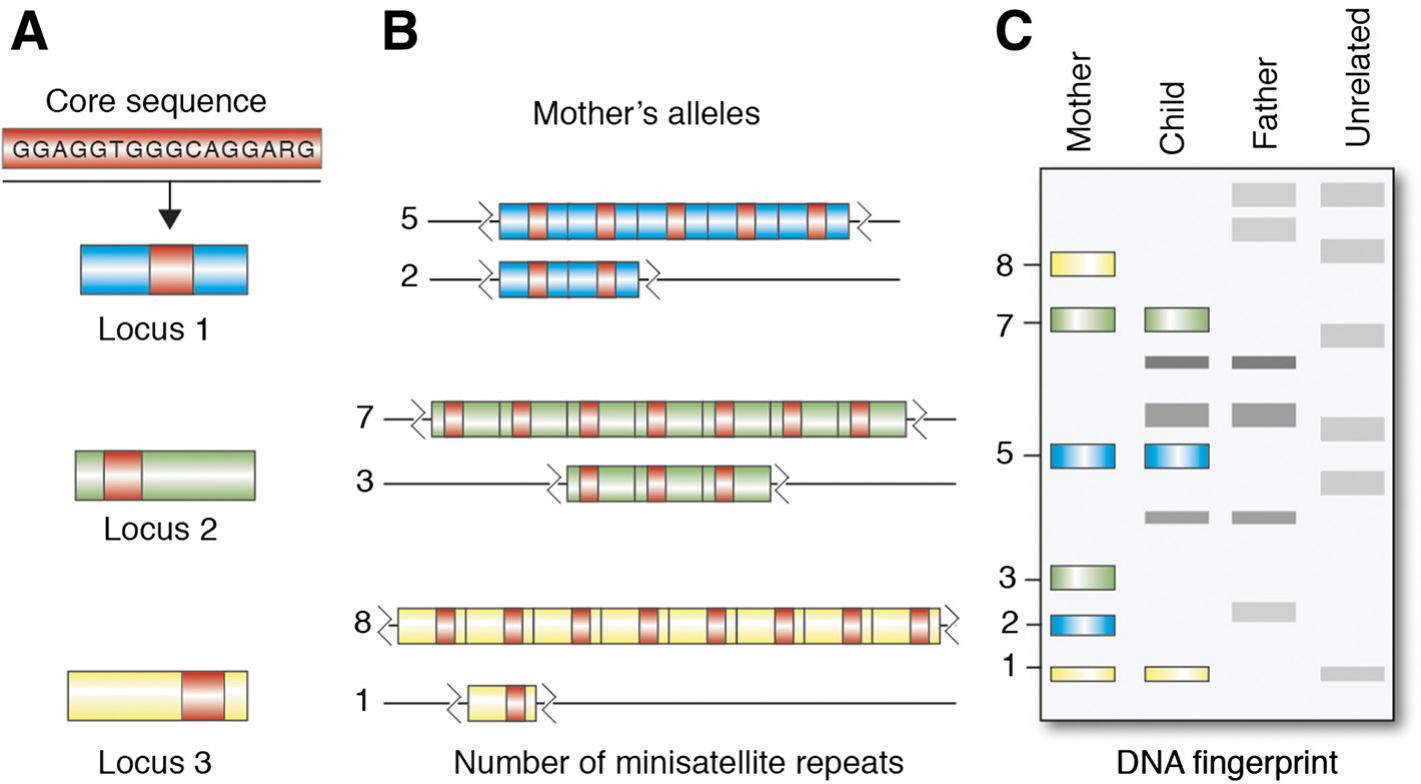
**Minisatellite repeat units are characterized by an approximate 16 bp core sequence in humans and other animals. (A)** A core minisatellite repeat is present at three loci. **(B)** The number of minisatellite repeats at these loci are shown for one individual (the mother) who is heterozygous at each of the three loci. Locus 1 genotype: 5, 2; locus 2 genotype: 7, 3; and locus 3 genotype: 8, 1. **(C)** Representation of an autoradiograph showing restriction fragment profiles of four individuals at these three loci. At each locus in the child’s profile, one allele is shared with the mother and the other is shared with the father, as would be expected when maternity and paternity have been correctly identified. Note that the unrelated individual shares only a small number of bands with the individuals from this family.

The first protocols for visualizing multilocus DNA fingerprints [7] used relatively long ‘minisatellite’ (VNTR) DNA probes. Probes were hybridized to restriction enzyme-digested DNA that had been size-separated and bound to a nylon membrane. These early probes consisted of concatenates of short (approximately 16 bp) ‘core’ repeats that were found to be both highly conserved and also distributed throughout the genome [3] (Figure 1). These conserved core regions were found within the highly repetitive minisatellite repeat sequences. Some of the most commonly used probes were derived from an intron of myoglobin, and were referred to as ‘33.15’ (consisting of 29 repeats of a minisatellite core with 128 bp of flanking sequence) and ‘33.6’ (consisting of 18 repeats of a 37 bp sequence unit. The 37 bp sequence unit comprised three repeats of a 11 to 12 bp core plus two base pairs) (sourced from Jeffreys’ United States Patent: US5413908; http://www.google.com.au/patents/US5413908).

**Figure 2:**
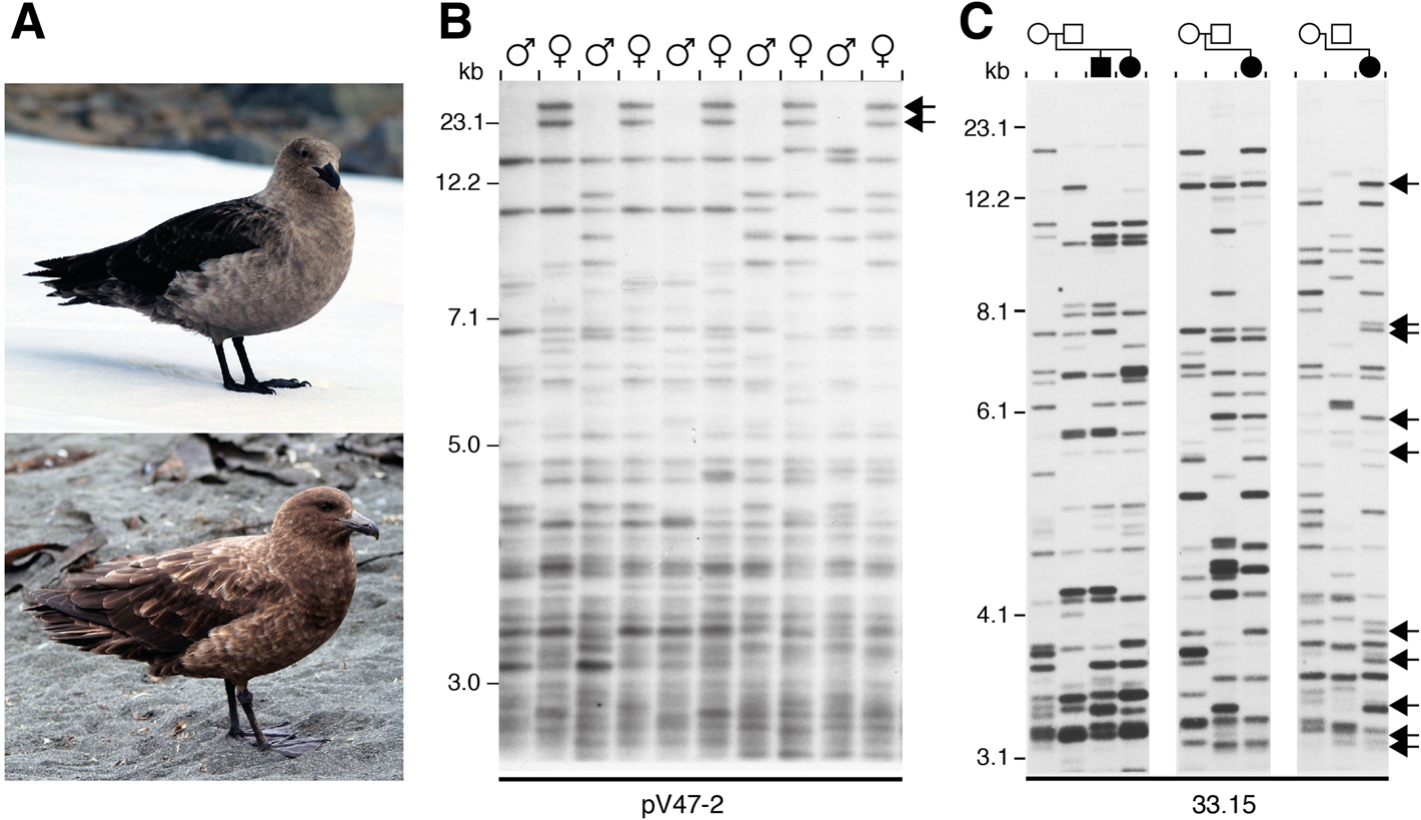
**Sexing and paternity in skuas. (A)** An adult south polar skua (*Catharacta maccormicki*; above) and an adult brown skua *(C. lonnbergi; below).***(B)** Multilocus DNA fingerprints resulting from hybridization of probe pV47–2 to genomic DNA from male and female brown skua digested with the restriction enzyme *Hae*III [14]. Arrows indicate two sex-linked DNA fragments that are present in females but absent in males. **(C)** Multilocus DNA fingerprints of three south polar skua families with the proposed relationships indicated above. DNA fragments that cannot be attributed to either putative parent (resident at the nest) are indicated by arrows.

Multilocus, minisatellite probe analysis (early ‘DNA fingerprinting’) required relatively large (that is, microgram) amounts of high quality, high molecular weight, genomic DNA digested with an appropriate restriction enzyme. Restriction enzymes with 4 bp recognition cut sites, such as *Hae*III, were commonly used because they cleave DNA frequently and hence typically digest the target DNA sequences close to the repeat motifs. The resulting fragments were separated according to size by electrophoresis through agarose gels, and then transferred to a nitrocellulose or nylon membrane in preparation for Southern blot hybridization (see Kirby [17] for detailed descriptions). The membrane containing denatured DNA fragments was then hybridized to a radioactively labeled copy of the minisatellite DNA probe (that is, concatenates of 16 bp minisatellite ‘core’ repeats). Hybridization of the labeled minisatellite probes to the digested DNA was detected by autoradiography (although additional labeling methods were sometimes used, including those based on the detection of light using horseradish peroxidase). Successful multilocus minisatellite hybridization typically produced a unique pattern of signals, ‘a DNA fingerprint’, of co-dominant markers that was unique to an individual (Figure 2). When two parents and an offspring were analyzed, a clear pattern of inheritance was usually observed (Figures 1 and 2C).

For zoologists, the difficulty in applying this technique was the requirement for specialized molecular biology skills, at the time not generally available to many in the field. The Southern blotting technique is a lengthy and precise method where well-designed experiments and careful benchwork are necessary for optimal results [18]. Many early DNA fingerprinters experienced non-specific probe hybridization problems with blotting media and resorted to the inclusion of one or more exotic blocking agents, such as dried milk powder, in pre-hybridization buffers. Furthermore, probes were not conveniently available from scientific biotechnology companies and had to be propagated in cloning vectors. Labeling these probes required ^32^P radioisotope facilities and expertise. Additionally, one could never be certain ahead of time how long to leave autoradiographs to develop in order to obtain the clearest signals.

With experience, many laboratories were able to produce high quality multilocus minisatellite profiles. Regrettably, this is where many of the more difficult technical problems began. In order to compare between gels, a ‘standard’ individual of known DNA concentration was used. By varying conditions it was possible to ensure that the same number of bands were present in this individual, on different gels. Restriction fragments could then be unequivocally recorded as present or absent (coded as 1, 0 in a data matrix; see Kirby [17] p. 240). In practice, however, some signals were very weak, making scoring difficult. This problem was not helped by the non-linear response of autoradiography film to exposure time. Similarly, it was often difficult to determine if signals of similar mobility in lanes widely separated across a gel had truly migrated the same distance from the origin. Statistical quantification of these results was relatively daunting and was usually calculated using a Mantel test to determine the correlation between two distance matrices.

**Figure 3:**
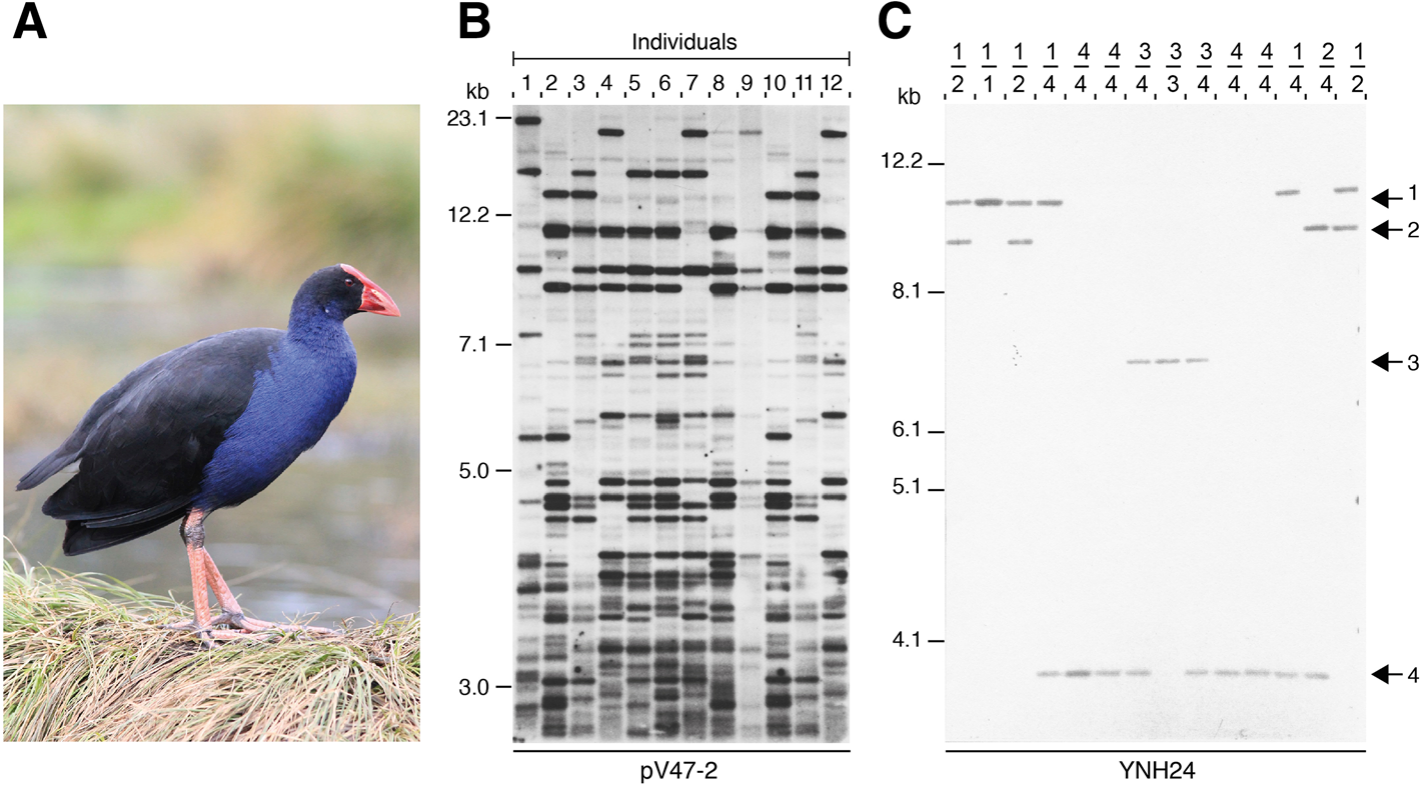
**Multilocus and single locus DNA fingerprinting in the pukeko. (A)** The pukeko or purple swamphen (*Porphyrio porphyrio*) is a communal breeder. **(B)** Multilocus DNA fingerprinting profiles of pukeko belonging to a communal group. Genomic DNA was digested with the restriction enzyme *Hae*III and hybridized to the probe pV47–2. **(C)** Single locus DNA profiles detected in pukeko using the minisatellite probe YNH24 [26]. Arrows indicated the four alleles detected and the genotype of each individual is given above.

A second generation of minisatellite DNA fingerprinting detected minisatellite repeat patterns at a single locus. One of two methods was used to detect these repeat sequences. First, in some cases, minisatellite probe hybridizations were performed under high stringency conditions with the probes sometimes hybridizing to unique, locus-specific stretches of sequences that flanked the repeat core. Successful hybridizations then resulted in simple single locus patterns, rather than the multilocus DNA profiles typically seen using the 33.6 and 33.15 probes under less stringent conditions (Figure 3C). A second approach involved the direct isolation of these locus-specific minisatellite sequences, although this method required significant effort. Genomic DNA libraries had to be constructed and subsequently screened with radioactively labeled core repeat probes to detect inserts containing useful polymorphic repeat DNA loci. The next step was to sub-clone the unique flanking regions of the repeat. These unique regions needed to be close enough to the repeat to be contained within a single restriction enzyme fragment. The fragment was then sub-cloned and used as a probe in Southern blots of genomic DNA. These fragments often returned the same simple patterns with only two co-dominant signals per individual.

Single locus minisatellite DNA fingerprints found immediate favor with forensic scientists, because these types of data were easier to defend as evidence in court. However, there were still fundamental questions to resolve in distinguishing alleles of the same apparent molecular size. Such alleles were distinguished through the use of ‘fixed bins’ (in which bins of particular molecular size ranges were *a priori* determined) or ‘sliding bins’ (these were not predetermined and were simply used to determine if signals were, within tolerance, similar in mobility) [19]. The simple patterns and hypervariable character of the probes, coupled with the fact that they could be used in combination to produce very high exclusion probabilities, quickly served to make them useful tools for forensic casework [20]. Early use of single locus minisatellites was largely limited to humans, as few zoologists were equipped to take up the technical challenge of creating panels of single locus probes for their favorite species; although the few who did are highlighted in the next section.

With the invention of the PCR an effort was made to incorporate the many advantages of this amplification method to the analysis of minisatellite loci. Jeffreys and co-workers developed this prospect with the development of a digital array typing technique [21,22]. This method was an impressive technological accomplishment, but its appeal to zoologists was limited. This was likely due to the advent of microsatellite-based methods that resulted in data which were technically much simpler to produce and easier to interpret, particularly for those with previous experience in allozyme electrophoresis and analysis. Nevertheless, Jeffreys’ early discoveries need to be credited for having led the way to the transformation of many researchers from ecology and evolutionary biologists into population and evolutionary geneticists.

### The introduction of DNA fingerprinting to experimental zoology

Jeffreys’ multilocus, minisatellite DNA fingerprinting methodology had its earliest, most significant impact on the study of avian mating systems [8,9]. Early publications challenged the ‘nuclear family’ model of birdlife where two doting parents raise their own exclusive offspring in a world of adversity. Costs and benefits of alternate breeding strategies, including brood parasitism and extra-pair copulation, were able to be investigated with new precision, and a number of studies probing kin selection models in a wide range of animals began to follow.

Gibbs *et al*. (1990) examined the dynamics of a spatially complex breeding colony of red-winged blackbirds (*Agelaius phoeniceus*). The dominant males sing and display strongly to secure the best (central) territories in the reed marsh. They attract most female partners to build nests there. They also gain most extra-pair copulations with females in neighboring territories, but this advantage is offset because females residing in their own territories gain more than average numbers of extra-pair copulations [23].

Owens *et al*. (1995) investigated the Eurasian dotterel (*Charadrius morinellus*), a species with sex-reversed plumage and polyandrous behavior. Here males guard the nest and provision young so they gain a ‘payoff’ only if they can be sure that the eggs that they look after are the products of their own gametes. Exactly how individuals would know this information is unclear. The investigators found that only ‘4.6% of chicks tested were not the genetic offspring of the caring male’. Hence, they were able to conclude that male dotterels succeeded in protecting their ‘parental investment’ via a ‘combined strategy of mate guarding and strategic timing of copulations’ [24].

Burke *et al*. (1987) provide an interesting contrast through their earlier study of the facultatively polyandrous songbird, the dunnock (*Prunella modularis*). In this species several males may accompany a single female. Males do not discriminate in favor of their own young, but provision the entire brood with an intensity of effort that reflects the amount of time that they had exclusive ‘access’ to the female just prior to egg laying [25]. The latter is then argued to represent a ‘reliable’ proxy for paternity.

Lambert *et al*. (1994) tackled an even more enigmatic case study, polygynandry in the pukeko (*Porphyrio porphyrio*). They found that supposedly dominant males did not consistently sire the majority of offspring in a group (Figure 3). These findings thus provide a serious challenge to conventional ideas about dominance and its presumed advantages [26]. Another species with a variable breeding system, including apparent female-female pairs, is the brown skua (*Catharacta lonnbergi*) [27]. In this case, DNA fingerprinting showed no extra-pair or extra-group breeding.

Other communally breeding birds have also been tested, including European bee-eaters (*Merops apiaster*) [28], Florida scrub-jays (*Aphelocoma coerulescens*) [29], and white-winged choughs (*Corcorax melanorhamphos*) [30]. In addition to a small central group of breeding adults, the tribe was found to consist mainly of offspring with delayed dispersal who were functioning as helpers at the nest. In the last of these three species members of one tribe may even ‘kidnap’ members of another to make up their numbers in an apparent effort to maximize foraging success. In contrast, the superb fairywren (*M. cyaneus*) also breeds co-operatively, but the majority of the offspring they tend are sired by males from outside the group [10]. In this case, the helpers assist the breeding female to offset the ‘costs’ of extra-pair fertilization.

Minisatellite DNA fingerprinting was applied to other avian breeding systems [31], to measure genetic variation, and to assess bird population structure to identify ‘source’ and ‘sink’ populations [32,33], for example in the blue duck (*Hymenolaimus malacorhynchos*). Minisatellite DNA fingerprinting was also used to determine genetic variability [34] and breeding behavior [35] in the critically endangered black robin (*Petroica traversi*) on New Zealand’s Chatham Islands. This species was shown to be socially and sexually monogamous, but with very low genetic variability, challenging conventional ideas about risk assessments associated with inbreeding in avian taxa. The black robin population suggested that once recessive deleterious alleles have been purged from bottlenecked populations by natural selection, the remaining individuals may be as fit as, or almost as fit as, comparable outbred populations. Only future events, such as a disease outbreak, will determine whether such genetically invariant species or populations are more vulnerable to extinction. However, in approximately 20 years since these studies were performed, the black robin continues to thrive with increasing numbers, despite the fact that the Chatham Islands still acts as a summer breeding ground for extremely large numbers of diverse avian species, resulting in the endangered black robin being exposed to an extraordinary diversity of pathogens.

Birds were not the only subjects of field studies using minisatellite DNA fingerprinting. For instance, Amos and colleagues carried out extensive work on mating systems in marine mammals [36,37]. In particular these were focused on testing if the energy costs of repeatedly fighting others to maintain dominance as ‘beachmasters’ was adequately repaid through exclusive ‘access to’ females and offspring sired. The lions (*Panthera leo*) of the Ngorongoro Crater in Tanzania provided an ideal opportunity for a similar study [38]. In this case young males have to choose (if they have the opportunity) between forming a large kinship guild with their brothers and pridemates (half-brothers or cousins) or a small guild with unrelated males. This takes place when they leave their home pride at maturity (or are forced out) and disperse in search of mates. Joining a large guild of other males increases the probability of making a successful takeover bid for mates in an already established pride of related females guarded by resident males. In guilds of related males the largest and most dominant male achieves the majority of copulations, but the unrelated males in the smaller guilds sire approximately equal numbers of offspring. These observations are in accordance with expectations from the Kin selection theory as in the larger guilds the sub-dominant males can be considered to have contributed to spreading their own genes through aiding the reproductive success of their dominant relative.

Minisatellite DNA fingerprinting was also used to examine population variation in other wild animals and in fish, examples include the California Channel Island fox (*Urocyon littoralis*) [39], the humpback whale (*Megaptera novaeangliae*) [40], and a number of commercial fish stocks [41]. Further applications for minisatellite analysis were also attempted, including the isolation of single locus probes for use in trait mapping and stock assignment [42-44], with mixed success. Jeffreys’ DNA fingerprinting methodology did, however, lead to the creation of some functional (though not always commercially viable) services, including animal paternity testing [45]. Commercialization of DNA profiling ventures for non-human subjects was difficult primarily due to the cost per test and time required to return results to clients. However these ventures did provide some beneficial services (for example the establishment of pedigrees may have prevented the loss of valuable breeding stocks [45]). In one unexpected case, an aging Afghan hound, thought to have zero viable sperm count, was shown to have sired a litter of pups in competition with a vigorous younger, less experienced, stud dog from the same kennel.

The Jeffreys research group applied their DNA fingerprinting techniques to a wide field in zoology. Work over several years on the mutational dynamics of minisatellites revealed many interesting aspects in primates [46] and rodents [47]. The Jeffreys’ research group also showed how DNA fingerprinting could aid captive breeding programs for rare and endangered species, such as the Waldrapp ibis [48]. Other tests were also developed directly from DNA fingerprinting methods, including assays for sex testing (Figure 2B) and assays for forensic wildlife services to control animal smuggling and illegal trade of endangered species [12,13].

### Later developments in DNA fingerprinting

Multilocus and single locus minisatellite-based ‘DNA fingerprinting’ methods were generally superseded by the use of single locus microsatellites to genetically identify individuals [49,50], and more recently by second generation sequencing (SGS)-based methodologies, including panels of SNPs. The original DNA minisatellite fingerprinting loci continued to be used to a limited extent for the characterization of individuals [51], population studies [52], the identification of disease markers [53,54] and quantitative trait loci, and the study of gene expression [55,56].

The transition to the use of single locus microsatellites as a method for ‘DNA fingerprinting’ was rapid, particularly in the USA, despite the fact that similar levels of technical laboratory expertise were required for both methodologies. Isolating species-specific DNA microsatellites, typically comprising of di-, tri-, and tetranucleotide repeat units, required substantial effort, particularly the construction and screening of genomic DNA libraries that were required to identify polymorphic loci. In some cases, however, the power of a multilocus assay using highly variable, generic minisatellite probes, succeeded in revealing differences between individuals when polymorphic microsatellite markers were unavailable or uninformative for a given species. An analysis of the kakapo (*Strigops habroptilus*), a nocturnal parrot endemic to New Zealand, illustrates this point particularly well [57].

Technical advances in microsatellite DNA fingerprinting, including rapid automated high resolution identification of alleles through capillary electrophoresis, simplified the procedure and increased its reproducibility. The use of PCR allowed genetic information to be recovered from trace amounts of modern and even ancient samples, and the repository of published PCR primers gradually expanded to include increasing numbers of species. Nonetheless, microsatellite-based methods are essentially based on the conceptual approach developed using multilocus, minisatellite DNA fingerprinting for the identification and characterization of individuals and populations. In this regard, the influence of DNA fingerprinting is likely to be an enduring one.

### Microsatellites in zoology

The disciplines of reproductive ecology and mating systems were markedly accelerated as the use of microsatellite-based DNA fingerprints became increasingly widespread. For example the application of DNA microsatellites to the offspring of vertebrates that simultaneously give birth to more than one offspring has revealed concurrent multiple paternities in a wide range of organisms, particularly in reptiles [58] (but also noted in birds and mammals). This phenomenon was first documented prior to the use of minisatellite DNA fingerprinting, for example in Belding’s ground squirrel [59] and eastern bluebirds using protein electrophoresis [60]. The technically less challenging aspects of isolating and routinely amplifying DNA microsatellites (compared with multilocus minisatellites) and their usefulness to unambiguously assigning parentage resulted in an exponential increase in studies of animal mating systems. Concurrent multiple paternity has now been widely documented in virtually all vertebrate groups investigated, for example it commonly occurs in over 50% of reptilian clutches [58]. Simultaneous multiple paternity has been detected in virtually all turtle species examined to date [61], lizards and snakes [62] (see summary in Uller and Olsson [58]), birds [63], and in mammals, where patterns of paternity have been studied [64]. In fish, where parentage has been studied quite extensively over the last two decades, microsatellite fingerprinting has revealed not only high levels of multiple paternity within broods, but also variable levels of multiple maternity as well, particularly in species with ‘male pregnancy’ or nest defence (reviewed by Coleman and Jones [65]). Multiply sired litters in mice were found to be relatively common (approximately 33 of 143 litters, or roughly 23%) in a survey of wild populations, with more prevalence in high density populations [66]. These observations, and the male postcopulatory competition (such as sperm competition) that may ensue [67], have fostered a great deal of speculation and literature surrounding the potential benefits of mating with more than one male. Hypothetical adaptive benefits have been proposed to include ensuring the complete fertilization of entire clutches of eggs [68,69], increasing the genetic variation of a litter [70], increasing the likelihood of successfully compatible gametes [71] (as reviewed by Dean *et al*. [66]), and the idea that mating with numerous males may serve as a type of confusion mechanism to decrease the probability of infanticide [72]. The ability to accurately assign paternity has provided a mechanism for testing of several hypotheses surrounding sperm competition, including the relative reproductive success and the significance of male mating order [73]. Numerous mechanisms of postcopulatory competition, such as embryonic cannibalism in sharks [74], have been suggested. Multiple matings by females have now been documented to occur with such frequency that the evolutionary significance of this phenomenon became the subject of considerable debate (see Wolff and Macdonald [72] for a review).

The use of DNA microsatellites has exposed other aspects of vertebrate mating systems that were initially considered to be rare anomalies. For example the first cases of virgin births (automictic parthenogenesis) in sharks were confirmed using DNA microsatellites [75-77]. Other vertebrates, including snakes [78] and lizards [79], have been documented to undergo parthenogenesis, including a high profile study documenting parthenogenesis in Komodo dragons [80]. Collectively these studies point to the existence of reproductive plasticity among females across a wide variety of taxa, and the consequences of this plasticity may be worthy of consideration for captive breeding programs involving endangered species [80]. Early examples of the genetic documentation of vertebrate asexual reproduction were limited to captive animals, but recent work suggests the existence of parthenogenesis also in wild litters of North American copperheads and cottonmouth snakes [81], suggesting that the phenomenon may be more widespread than previously thought.

### DNA microsatellites used to study population subdivision and male-mediated gene flow

DNA microsatellites have been useful tools describing population connectivity, isolation, and the particulars of inter-population gene flow. They have also been used extensively to assess population subdivision, sometimes in relation to geographical barriers [82]. Contrasting patterns of genetic partitioning between maternally inherited mitochondrial sequence data and autosomal DNA microsatellites have revealed levels of male-mediated gene flow in several species, including bats [83], great white sharks [84], sea turtles [85], sharks [86], and primates [87]; note this study also included Y chromosome markers.

### DNA microsatellites and conservation biology

Essentially, the extensive use of microsatellite DNA markers has directly given rise to a number of high profile journals, including *Molecular Ecology, Molecular Ecology Resources, and Conservation Genetics.* A very large number of microsatellite loci have now been used to document levels of genetic variation in rare and endangered species and thus better inform conservation management actions. In the Florida panther, for example, a small remnant population (less than 100 individuals) is thought to suffer from inbreeding depression. The population carries genetic anomalies including kinked tails and heart defects. In an attempt to mitigate inbreeding, several females from a Texas population were outcrossed with the Florida population. DNA microsatellites enabled researchers to generate detailed pedigrees to monitor the success of this genetic restoration program [88].

The application of DNA microsatellites has improved the ability to monitor and enforce conservation measures. As an increasing number of populations are characterized with DNA microsatellites they serve as useful databases to identify the provenance of animals confiscated at borders. Widespread progress has been made assigning various endangered and threatened species to geographic regions using DNA microsatellites, including tortoises [89], fish [90], bears [91], and elephants [92]. Although the existence of microsatellite databases is generally useful, problems do arise when genotype data are shared among laboratories, due to differences in allele scores for similar samples. This has been alleviated somewhat by the subsequent use of universal reference samples for allele calibration.

Although the processes by which microsatellites mutate are generally established, uncertainties persist about their mode of evolution, stepwise *versus* two-phase [93,94], as well as the extent with which these modes of evolution uniformly apply to repeats of varying motifs and sizes. Although these unknowns potentially compromise the application of standard population genetic statistics to microsatellite loci, most investigators treat them as if they were of minor influence. Some software bundles, such as Bottleneck [95], do account for different mutation models. Recent results show that human microsatellites have a predominantly stepwise mode of mutation, with a slight bias towards an increase in size and an upper size limit [96]. The same study also shows a higher mutation rate for tetranucleotide repeats than for dinucleotide repeats. This will allow investigators to concentrate on recovering suitable repeat types from genomic screens, as these not only promise to be more polymorphic but will also yield allelic genotypes that are easier to score. As a result, zoologists are now positioned for a new age of improved microsatellite studies supported by genomics and SNP analyses plus expression profiling to advance causal explanations for evolutionary phenomena.

### DNA fingerprinting in the era of whole genome second generation sequencing

Ecologists continue to develop microsatellite loci for population genetic studies using the relatively recently introduced SGS platforms [97], and pipelines are emerging to maximize the success rate of microsatellite PCR primer development from SGS runs [98-101]. Bioinformatics tools, including RepeatSeq [102] and lobSTR [103], are emerging to improve the mapping success of microsatellite reads from SGS data, although simple sequence repeats such as microsatellites remain relatively problematic to analyze with SGS (due to difficulties sequencing through the entire repeat, reliably allelotyping a locus, and complexities associated with bioinformatically mapping simple sequence repeats). One of the potential benefits of using SGS to analyze DNA microsatellite loci (fingerprinting) is the very high output of data. Additionally, deeper sequencing via SGS can reveal variant alleles that may go undetected when analyzed by PCR with fluorescently labeled fragments and capillary electrophoresis.

Will ecologists and zoologists continue to rely on DNA microsatellite fingerprinting of individuals and populations in the era of second and third generation sequencing? Panels of SNP loci analyzed on SGS platforms have a number of advantages over ‘traditional’ microsatellite and minisatellite fingerprinting, which may lead to their continued replacement over these VNTR-based DNA fingerprinting techniques in zoology. One advantage of using large panels of SNPs for fingerprinting is in their accuracy. However, in principle, although SNP variants can be unequivocally assigned to a single individual, analyses of non-invasively collected samples are also proving problematic because, like microsatellite loci, dropout of allelic SNPs can be significant [104]. An additional advantage is the requirement of only short stretches of DNA (<50 bp), allowing the analysis of highly degraded material such as that found in environmental samples (such as soil), low quality non-invasive samples (such as feces), as well as historical and ancient DNA. Research along these lines has allowed association and evolutionary studies of a number of iconic animals, including 40,000-year-old DNA from a wooly mammoth [105], 120,000-year-old DNA from a polar bear [106] and, very recently, a 700,000-year-old horse genome [107].

Rapidly developing SGS technologies now make it possible to obtain ‘complete’ animal genomes in less than a day, that in the near future will no doubt be affordable for most laboratories. At present, the sequencing capacity of larger second generation sequencers, such as the HiSeq 2500 (Illumina, San Diego, CA, USA), is up to 600 Gb (gigabases) for a full run (reviewed by Glenn [108]) and about 250 Gb for the more affordable Ion Proton. The latter, when equipped with a PIII sequencing chip, promises to deliver high coverage of complete animal genomes in less than a day for less than USD$1,000 [108].

Data from a complete nuclear genome sequence is absolute. In addition to ‘all’ microsatellite and minisatellite sequences, complete genomes provide information regarding SNPs, insertions/deletions, as well as any genome rearrangements that would be difficult to detect using fragment length-based analysis such as capillary electrophoresis (microsatellites) or probe hybridizations (minisatellites).

For modern genomes, third generation sequencers are likely to be of even greater use. The ability of these single DNA strand sequencers to sequence strands up to 100,000 bases long in very short times will provide not only information on sequence variation but also on linkage. At present these sequencers suffer from high error rates, approaching 15%, but their utility is in the assembly of animal genomes, thereby clarifying possible linkages between SNPs and/or sequence repeats (see Weaver [109] and Ozsolak [110] for reviews).

The existence of large microsatellite profile databases has served to benefit many population and ecological studies. In the same way the accumulation of large computer databases of animal genomes will eventually benefit future molecular ecology studies. The collection, storage, and maintenance of a complete genome database, however, will no doubt invoke a number of storage issues. Storage of animal genomes, although a few gigabases in size, actually contain much less ‘usable’ sequence, particularly if only SNPs (identified by comparison with a suitable reference genome) are used. As the average number of SNPs in a genome is 1 per 1,000 bases, this would effectively require less than 30 megabytes (MB) of storage. This translates to a total of approximately 30,000 terabytes (TB) of required storage for the SNPs of 1 billion animals, storage that is available today. With the information inherent in genome-wide SNPs, DNA fingerprinting, born from Jeffreys’ initial discovery of minisatellites, is likely to continue in the near future with the use of ‘complete’ genome datasets.

## Conclusions

It is clear that minisatellite DNA studies of humans and other animals were the successful forerunners of today’s microsatellite DNA genotyping methods. But, because minisatellite DNA methods employed Southern blot analyses, these were both time-consuming and technically challenging to perform on a regular basis. In addition, minisatellite DNA analyses required high quality and large amounts of sample DNA, which diminished the usefulness of this technique for ancient and/or degraded samples. In contrast, microsatellite DNA amplifications by PCR detected similar, although shorter, repeat sequences and could be applied to ancient and lower quality samples. Hence, the use of DNA microsatellites became more widespread among researchers than the minisatellite-based fingerprinting systems ever were. Nonetheless, Jeffreys’ original insight, that repeated minisatellite DNA sequences could be used to study a range of zoological issues, represents one of the important technical and intellectual achievements in the history of zoology.

## Abbreviations

PCR: Polymerase chain reaction; SGS: Second generation sequencing; SNP: Single nucleotide polymorphism; VNTR: Variable Number Tandem Repeat.

## Competing interests

The authors declare that they have no competing interests

## Authors’ contributions

GKC wrote the first draft of the paper after initial discussion with the other authors. GKC, CC, and LH collected the reference list and all authors contributed to subsequent drafts. CDM and DML designed and contributed the figures. All authors read and approved the final manuscript.
